# *APOL1* Null Alleles from a Rural Village in India Do Not Correlate with Glomerulosclerosis

**DOI:** 10.1371/journal.pone.0051546

**Published:** 2012-12-26

**Authors:** Duncan B. Johnstone, Vijay Shegokar, Deepak Nihalani, Yogendra Singh Rathore, Leena Mallik, Ashish, Vasant Zare, H. Omer Ikizler, Rajaram Powar, Lawrence B. Holzman

**Affiliations:** 1 Renal-Electrolyte and Hypertension Division, University of Pennsylvania, Philadelphia, Pennsylvania, United States of America; 2 Department of Microbiology, Government Medical Hospital, Nagpur, Maharastra, India; 3 CSIR- Institute of Microbial Technology, Chandigarh, India; 4 Public Health Institute of Nagpur, Maharashtra, India; Fondazione IRCCS Ospedale Maggiore Policlinico & Fondazione D'Amico per la Ricerca sulle Malattie Renali, Italy

## Abstract

**Background:**

Among African-Americans, genome wide association revealed a strong correlation between the G1 and G2 alleles of *APOL1* (apolipoproteinL1, also called trypanolytic factor) and kidney diseases including focal and segmental glomerulosclerosis, HIV-associated nephropathy and hypertensive nephrosclerosis. In the prevailing hypothesis, heterozygous *APOL1* G1 and G2 alleles increase resistance against Trypanosoma that cause African sleeping sickness, resulting in positive selection of these alleles, but when homozygous the G1 and G2 alleles predispose to glomerulosclerosis. While efforts are underway to screen patients for G1 and G2 alleles and to better understand “*APOL1* glomerulopathy,” no data prove that these *APOL1* sequence variants cause glomerulosclerosis. G1 and G2 correlate best with glomerulosclerosis as recessive alleles, which suggests a loss of function mutation for which proof of causality is commonly tested with homozygous null alleles. This test cannot be performed in rodents as the *APOL* gene cluster evolved only in primates. However, there is a homozygous *APOL1* null human being who lives in a village in rural India. This individual and his family offer a unique opportunity to test causality between *APOL1* null alleles and glomerulosclerosis.

**Methods and Findings:**

We obtained clinical data, blood and urine from this *APOL1* null patient and 50 related villagers. Based on measurements of blood pressure, BUN, creatinine, albuminuria, genotyping and immunoblotting, this *APOL1* null individual does not have glomerulosclerosis, nor do his relatives who carry *APOL1* null alleles.

**Conclusions:**

This small study cannot provide definitive conclusions but the absence of glomerulosclerosis in this unique population is consistent with the possibility that African-American glomerulosclerosis is caused, not by loss of *APOL1* function, but by other mechanisms including a subtle gain of function or by the “genetic hitchhiking” of deleterious mutations in a gene linked to *APOL1* G1 and G2.

## Introduction

Compared to European-Americans, African-Americans have a 3–4 fold higher rate of incident and prevalent end-stage kidney disease, including a higher rate of diabetic nephropathy, focal and segmental glomerulosclerosis (FSGS), HIV-associated nephropathy (HIVAN), and the ill-defined entity of “hypertensive nephrosclerosis [1].” Using mapping by admixture linkage disequilibrium, two groups in 2008 reported that FSGS, HIVAN and hypertensive nephrosclerosis in African-Americans correlate with single nucleotide polymorphisms (SNPs) on Chromosome 22q within the gene *MYH9*, which encodes non-muscle myosin heavy chain 2A [2], [3]. *MYH9* was an excellent candidate disease gene at this locus for several reasons, the foremost of which is that rare autosomal dominant mutations in *MYH9* can cause glomerulosclerosis in patients with Epstein's Syndrome and Fetchner's Syndrome, in which patients usually reach end stage kidney disease during their 3^rd^–4^th^ decade of life [4], [5]. Based on additional GWAS findings in other populations [6], [7] and additional data, investigators proposed a distinct entity of *MYH9*-glomerulosclerosis [8]–[10]. However, a causal mutation in *MYH9* remained elusive, as did an explanation for why these common, intronic *MYH9* polymorphisms were present at such high frequency in African-American and West African populations, and an explanation for why the majority of African-Americans with 2 copies of these polymorphisms were unaffected by kidney disease. Deletion of *Myh9* from podocytes in experimental mice resulted in a predisposition to glomerulosclerosis in response to injury, which mimicked the predisposition to kidney disease among African-Americans with *MYH9* “risk alleles [11],” but the uncertainty of *MYH9* as the causal gene for African-American glomerulosclerosis remained.

In July of 2010, new data from the 1000 s Genomes Project led two groups to conclude that *MYH9* is not the relevant disease gene for African-American glomerulosclerosis. Both groups found that African-American glomerulosclerosis correlates more strongly with the G1 and G2 alleles of *APOL1*, which lies immediately centromeric to *MYH9* [12], [13]. When *APOL1* G1 and G2 alleles are accounted for, the strength of the correlation between *MYH9* SNPs and African-American kidney disease is either significantly reduced [13], [14], or effectively eliminated [12] due to linkage between *APOL1* and *MYH9*. In addition, analysis of the worldwide distribution of *MYH9* polymorphisms demonstrated that the high allele frequency of the *MYH9* ”risk haplotype” among African-Americans and West Africans could be accounted for by linkage with a nearby site of selection, such as within *APOL1* [15]. Based on these and other data, *APOL1* supplanted *MYH9* as the candidate disease gene for glomerulosclerosis and hypertensive nephrosclerosis among African-Americans. Reports of the correlation between *APOL1* and glomerulosclerosis prompted the development of genetic tests for the G1 and G2 alleles [12], the proposal of “APOL1 glomerulosclerosis” as a distinct disease entity [16], and the suggestion that selected African-Americans might benefit from screening for G1 and G2 alleles including potential kidney donors and asymptomatic HIV+ patients as an indication for early initiation of HAART [17]. However, *APOL1* may not be the gene responsible for African-American glomerulosclerosis at this locus on Chr22. While this is an initial caveat of all discoveries from genome wide association studies due to linkage [18], such caution is not currently stressed amidst the momentum of *APOL1*-glomerulopathy. For this reason, we feel it is necessary to examine the strengths and weaknesses of *APOL1* as a candidate gene for African-American glomerulosclerosis with an extended introduction in order to justify the aims of our study.

In contrast to *MYH9*, *APOL1* can explain why the alleles proposed to cause glomerulosclerosis are found at such high frequency in the African-American population. *APOL1* was identified as a protein principally made by the placenta, endothelium and liver that is secreted into serum and circulates as a transmembrane component of HDL_3_ lipoproteins [19], [20]. The only known biological function for *APOL1* at this time is as the key component of human Trypanolytic Factor (TLF), which kills blood parasites such as *T.b.rhodesiense*, the agent of acute African sleeping sickness [21], [22]. The *APOL* gene cluster arose about 70 million years ago only in primates and has been under significant positive selection [23], as is seen with pathogen resistance loci. *APOL1* evolved about 30 million years ago in primates and is only expressed in humans, gorillas, baboons and possibly a few other primates [24]–[26]. In response to *APOL1*, the serum resistance associated gene (*SRA*) evolved in *T.b.rhodesiense*. The SRA polypeptide binds the C-terminus of APOL1, preventing its anion conductance and lytic activity and allowing Trypanosoma to survive [27], [28]. Last to evolve were the *APOL1* G1 and G2 alleles, which alter the SRA binding site on APOL1 without inactivating its anion conductance, thereby restoring APOL1 lytic activity [12], [29]. In a study of the distribution of the G1 and G2 alleles in most major human populations (with a notable exception of the Indian subcontinent) the G1 and G2 alleles are present only in sub-Saharan African populations and African-Americans, with a particularly high allele frequency of G1 among Yorubans in Nigeria [30]. A small geographical mystery exists, because SRA-expressing trypanosomes currently are found only in eastern/southeastern Africa rather than Nigeria/West Africa, but it is possible that trypanosome watersheds have changed over time, or that the G1 and G2 alleles also confer resistance to other blood-born parasites endemic to West Africa. Mystery aside, an attractive hypothesis analogous to sickle cell anemia emerged in which the disadvantage of adult-onset glomerulosclerosis when homozygous for G1 and G2 is offset by the advantage of resistance to infection by SRA-expressing Trypanosomes and possibly other parasites, affording a survival advantage to heterozygotes [12], [13]. This survival advantage is consistent with the evidence that the *APOL* subfamily has been a target of positive selection over evolution [23].

This hypothesis can be examined from both sides: the heterozygous advantage of parasite resistance and the homozygous disadvantage of kidney disease. The G1 and G2 alleles (2 linked missense variants for G1, and a 6 bp in-frame deletion for G2) alter residues in the SRA binding domain in the C-terminus of APOL1 [12], [13], and this SRA-binding domain can be deleted without inactivating APOL1 lytic activity, [21], [22], so the G1 and G2 alleles *should* confer resistance to SRA-expressing Trypanosome infection. While there is no evidence for this advantage *in vivo* (for instance, a near-absence of G1 and G2 has not yet been reported from a cohort of patients with Trypanosomiasis), the *in vitro* data are compelling [12]. Trypanosomes lacking SRA are killed when incubated in any human sera, whereas Trypanosomes that express SRA are 100% resistant to incubation with wild-type serum but are completely lysed when incubated with G1 or G2 serum. Similarly robust lysis against SRA-expressing Trypanosomes was demonstrated with purified, recombinant G1 or G2 variants of APOL1. Moreover, lysis occurred with a similar dose-response curve using serum from either heterozygous or homozygous individuals, even when diluted 100X or less, suggesting a heterozygote should have more than enough APOL1 lytic activity to resist infection.

On the other hand, the evidence for a homozygous disadvantage of glomerulosclerosis due to two copies of G1 and G2 is much less clear. While there is an excellent *correlation* between the G1 and G2 alleles and African-American glomerulosclerosis, there are no data demonstrating that the G1 and G2 sequence variants *cause* glomerulosclerosis, or by what mechanism, and there are several incongruities that complicate the story. First, the magnitude of the effect of *APOL1* G1 and G2 on the risk of glomerulosclerosis is sufficiently unusual that these alleles were proposed to occupy a unique niche in human disease [31]. Using the most recent data, the Odds Ratio for glomerulosclerosis from 2 alleles of G1 or G2 in a recessive model is 16.9 for FSGS (confidence interval 11–26.5) and 29 for HIVAN (c.i. 13.1–68.5) [30], which is incredibly high compared to the effect of all previously described disease-*modifying* genes identified by GWAS, which usually confer a small Odds Ratio of 1.3–1.6. On the other hand, the lifetime attributable risk of idiopathic glomerulosclerosis for people homozygous for the G1 and G2 alleles has been estimated at only ∼5% [30], and compared to disease-*causing* mutations this phenotypic penetrance is extremely low. Various theories for this atypical phenotypic penetrance have been put forth and the most persuasive is that, similar to initial theories for *MYH9* and African-American glomerulosclerosis, perhaps the *APOL1* G1 and G2 alleles do not cause but rather predispose to kidney disease, and a second gene-gene or gene-environment interaction (as yet unknown) is required for the phenotype. A gene-gene interaction appears increasingly unlikely, as the previously unsuccessful attempts to find second-site loci for the *MYH9 E1* haplotype should apply similarly to *APOL1* since the loci are linked and the cohorts of patients are nearly identical. Similar efforts undertaken more recently to control for *APOL1* and look for second sites of significant association with kidney disease also have not been fruitful. A gene-environment interaction such as a viral co-infection is possible, as suggested by the extremely high Odds Ratio of G1 and G2 alleles with HIV-associated nephropathy [30]. However, the virus that might interact with *APOL1* to cause the more common idiopathic focal and segmental glomerulosclerosis has not been discovered, nor has a mechanism been uncovered for the proposed interaction between *APOL1* and HIV or any other virus that would explain why all forms of glomerulosclerosis arise with the G1 or G2 alleles but not with the wild type *APOL1* allele. In short, a viral-gene interaction is a conceivable explanation for the atypical penetrance of G1 and G2 alleles, but there are no data to support this at present.

Secondly, assuming that *APOL1* is the relevant disease gene, there are no data on the mechanism by which the G1 and G2 alleles of *APOL1* (and not the wild type allele) might cause glomerulosclerosis. Suggested mechanisms by which the G1 and G2 variants cause glomerulosclerosis include apoptosis, autophagy, lipoprotein-mediated cell signaling, and oxidative stress within the glomerulus [12], [13], [30], [31]. Autophagy remains the most attractive hypothesis because APOL1 contains a 9-amino acid BH3 domain, and other members of the Bcl2 family that contain only a BH3 domain do not protect against cell death but rather activate apoptosis or autophagy in response to cellular stresses [32]. When overexpressed in cultured gastrointestinal epithelia, *APOL1* activates type II autophagy via LC3 resulting in cell death [33]. However, the cultured cells in these studies do not express endogenous *APOL1*, and it is not yet clear whether the autophagy observed in this model system occurs in cells that normally express APOL1 *in vivo*. Additional data that would support a connection between *APOL1* alleles and podocyte cell death could include evidence that the endogenous gene is necessary for autophagy, in which an external stimulus activates autophagy that is prevented by knockdown or inhibition of endogenous *APOL1*. Absent such data, alternative explanations for the observation of autophagy in cultured gastrointestinal cells transfected with *APOL1* include a toxicity of APOL1 on cells that normally do not express this protein, toxicity due to heterologous overexpression of any BH3-only protein, or a non-specific toxicity due to heterologous protein overexpression in cultured cells, as has been observed with many integral membrane proteins including ion channels and G-protein coupled receptors [34]. Finally, whether implicating autophagy or any other mechanism mentioned above to explain glomerulosclerosis, one must explain why this occurs only with the G1 and G2 variants and not with wild type *APOL1*. In short, while G1 and G2 may cause glomerulosclerosis through increased autophagy, there are very little data in support of this at present.

Third, if *APOL1* is assumed to be the relevant disease gene, the site of action is not yet clear. The G1 and G2 alleles could cause glomerulopathy as a circulating serum component, as an endogenously expressed protein within the kidney, or more precisely in a cell-autonomous fashion from endogenous podocyte expression. This latter idea forms the basis of the prevailing hypothesis for the G1 and G2 alleles: that *APOL1* is expressed within podocytes, that the G1 and G2 alleles inappropriately activate autophagy within podocytes, and that the resulting death of podocytes results in glomerulosclerosis. APOL1 staining recently was characterized for the first time in adult kidneys, including normal tissue and biopsies from patients with FSGS and HIV nephropathy, and the authors reported several surprises [35]. APOL1 staining was observed within podocytes, however, definitive conclusions are difficult as we respectfully suggest that the podocyte staining was quite faint in comparison to tubular staining, and in some cases the podocyte staining was patchy, consistent with a protein that is not endogenously expressed but is absorbed by podocytes after glomerular filtration (because filtration is not uniform along the glomerular capillary). Moreover this podocyte staining disappeared from glomerulosclerotic biopsies including G1 and G2 positive kidneys, which runs counter to the prevailing hypothesis that G1 and G2 might be causing an excessive autophagy signal in podocytes.

In summary, if the G1 and G2 alleles of *APOL1* cause kidney disease, then there are several questions for which we have insufficient answers, including a mechanism of injury due to the G1 and G2 variants but not wild type APOL1, and an explanation for why up to 95% of people with two copies of G1 or G2 alleles do not develop FSGS. Answering these questions in order to firmly establish causal proof of the disease gene for African-American glomerulosclerosis will be both difficult and important. In this study we use classical genetic inference, starting from the premise that the G1 and G2 alleles of *APOL1* correlate with glomerulosclerosis best in a recessive model, , with a similar risk for FSGS in homozygous or compound heterozygous individuals (G1/G1, G2/G2, or G1/G2), with an Odds Ratio that ranges from 6.7 up to 16.9 in the most recent study [12], [13], [30]. In initial reports, a small but statistically significant correlation was reported for glomerulosclerosis with a single allele of G1 or G2, suggesting a potential dominant effect (or what is sometimes termed a semi-dominant effect, in which one allele results in a phenotype but two alleles result in a stronger phenotype); in support, one of the subsequent analyses of the *APOL1* G1 and G2 alleles found a risk of glomerular disease that fit a semi-dominant or additive model [13], [36]. A dominant phenotype can arise via a number of different molecular mechanisms, including a gain-of-function, a gain-of-new-function, haploinsufficiency, dominant negative, loss of heterozygosity and more [37], [38] and therefore definitively testing the underlying molecular mechanisms by which *APOL1* G1 and G2 alleles might cause kidney disease would be complex and arduous if the phenotype were semi-dominant. However, researchers recently pointed out that by virtue of the proximity of G1 and G2 to each other they are in almost complete negative linkage disequilibrium. Therefore, in order to calculate the effect size of a single G2/+ allele rather than the combined effect of G2/+ and G2/G1, one should stratify by the presence or absence of the other allele [39]. This stratification was performed recently in concert with a larger sample size of G1 and G2 positive patients with idiopathic and HIV-related glomerulosclerosis and the previously observed dominant effect abated (Odds Ratio 1.41, c.i. 0.8–2.6) [30], with the effect strongly fitting a recessive pattern of inheritance, similar to the original analysis by Genovese et al, 2010. In addition to the risk of FSGS in native kidney disease, a small study that is worthy of replicating found that early allograft failure correlated with G1 or G2 alleles in the donor kidney, with a hazard ratio of 3.84 in a purely recessive model (donors with 2 alleles compared to 0/1 alleles) [40], [41]. A recessive pattern of inheritance simplifies classical genetic inference because the overwhelming number of recessively inherited traits result from loss of function mutations. Therefore, if an *APOL1* canonical loss of function mutation (a null allele) predisposes to glomerulosclerosis, then *APOL1* loss of function is the likely mechanism of glomerulosclerosis in individuals with African-ancestral G1 and G2 alleles. Testing a null allele would usually be performed by generating a germline mouse knockout in the laboratory, or through random mutagenesis in a non-complementation screen in other model organisms. However, the *APOL1* gene is not present in experimental animals, so neither approach is feasible.

Fortuitously, there is one previously described human being who is null for *APOL1*, and this person and his family can provide a test for the relationship between canonical *APOL1* loss of function and kidney disease. This individual lives in a rural village in central India and came to the attention of medical authorities after becoming critically ill due to an infection with *T. evansi*, a Trypanosome endemic to Water Buffalo. While his fascinating clinical course has been previously described in the infectious disease literature, including the identification of his *APOL1* null alleles [42], [43], there are no reports describing whether or not he has kidney disease. To return to classical genetic inference, if the G1 and G2 alleles predispose to kidney disease in a recessive model, which suggests a loss of function mutation, then the presence of glomerulosclerosis in this *APOL1* null gentleman would support this model using a “gold standard” loss-of-function allele. However, if this individual does not have glomerulosclerosis this would support (but would not prove) an alternative hypothesis: that the cause of kidney disease is not due to recessive inheritance of *APOL1* G1 and G2 themselves, but rather something that is linked to *APOL1* G1/G2. The previously reported haplotype blocks for G1 and G2 alleles are quite large [12], with the consequence that a large number of polymorphisms co-segregate with G1 and G2, including sequence variants in nearby genes and potential cis-regulatory elements that could be alternative candidates for a kidney disease “causal mutation.” This forms the basis of our hypothesis that, during positive selection for the G1 and G2 alleles among sub-Saharan populations due to the benefit of Trypanosome resistance, one or more sequence variants in the vicinity of *APOL1* “hitchhiked along,” and it is these linked sequence variants that cause glomerulosclerosis. Similar examples of this type of inheritance have been described recently in the literature, including Crohn's disease and Marfan's syndrome, in which positive selection of an allele that improves nutrition or disease-resistance leads to unintended inheritance of linked and potentially deleterious sequence variants due to “genetic hitchhiking” [44], [45]. Herein we describe a collaborative effort of researchers from the USA and India to test this *APOL1* null gentleman from a small rural village in central India as well as his family and a subset of his fellow villagers for the previously described *APOL1* null alleles, for APOL1 protein expression, and for clinical evidence of glomerulosclerosis.

## Results

The source patient lives in Seoni village, a remote, traditional Indian village approximately 3–4 hours drive from Nagpur in eastern Maharashtra. A collaborative team of investigators from the USA and India visited Seoni village after obtaining IRB approval and permission from local government and police officials. Logistical difficulties included obtaining informed consent in Marathi, initial processing of biological samples in Nagpur, and obtaining clinical tests that are not routinely performed in the locale.

After obtaining informed consent, clinical data and biological material were obtained from the source patient and 50 members of his family and his village. Per guidelines of the Indian Government and the Indian Council of Medical Research, all studies of biological materials of Indian citizens were performed within India and no biological material was exported. To screen for evidence of focal and segmental glomerulosclerosis (FSGS) and other kidney diseases, de-identified participants provided clinical data to Marathi-speaking physicians including Past Medical History, Family History of kidney disease or unexplained sudden death as a potential proxy for undetected end stage kidney disease in this rural setting, and sufficient family information to construct a family dendogram. Blood pressure, which is often elevated in chronic kidney disease, was measured by manual sphygmomanometry. Blood samples were obtained for clinical tests of kidney function, for DNA sequencing, and for measurement of APOL1 in serum and urine by immunoblotting. Urine was acquired by clean catch in order to obtain a urine albumin∶creatinine ratio.

**Figure 1:**
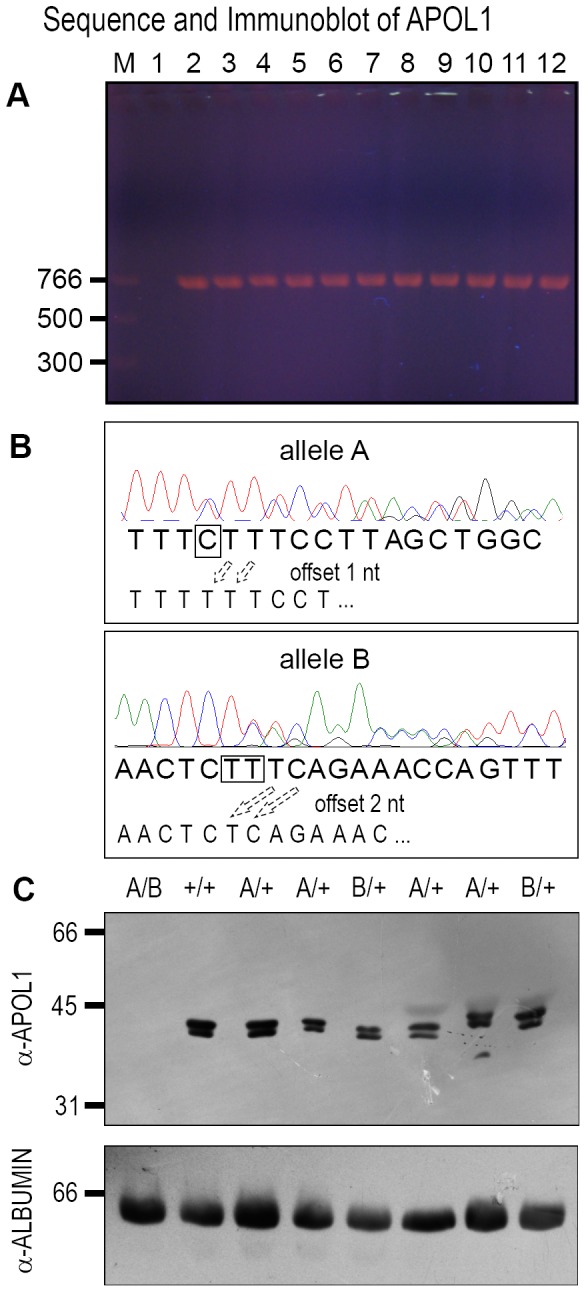
*APOL1* null alleles in 51 participants. (A) PCR products that span alleles A and B were amplified from participant genomic DNA and yielded a product of the expected size. Lane M = 5 uL Quickload PCR marker. Lanes 1–12 used the same master mix. Lane 1 is water control and lanes 2–12 have 100 ng genomic DNA from different participants. (B) Sanger sequencing identified alleles A and B. Purified PCR products were sequenced with nested internal primers. Sequence tracings are shown for instances in which allele A or allele B was detected. As shown, signal interpretation is clear until the site of the deletion, after which the tracing shows a mixture of two signals. Half of the signal is from the wild type allele, with the expected nucleotide as shown below, and half the signal is from the nucleotide offset by either 1 nt (allele A) or 2 nts (allele B). Additional tracings are available in supplemental data (C) Immunoblots of serum from all 51 participants were probed with α-APOL1. Loading controls used α-albumin as shown or α-transferrin (not shown). M = marker. Lanes 1–8 are participant serum, with a doublet of ∼41/39 kDa observed in all lanes except one, which corresponds to the null patient. These blots were confirmed in triplicate.

For each of the 51 participants, PCR was used to amplify a product of the expected size that spanned the two previously identified nonsense alleles from the source patient (Fig. 1A) [43]. We refer to these alleles using the previously published designations: “allele A” is a single cytosine deletion within the sequence AACTTT**C**TTTCC and “allele B” is a deletion of two adenines within the sequence TTTCTGA**AA**GAGTT [43]. Using nested primers, both strands were analyzed by Sanger sequencing for each patient. Nonsense alleles A and B were confirmed with at least 2 primers from different directions and were easily identified as a transition from a clear sequence signal to a mixed signal in which the wild type sequence is admixed with the sequence offset by the 1 or 2 nucleotide deletion from allele A or B, respectively (Fig. 1B, and supplementary Fig. S1). In addition to sequence analysis, immunoblots were performed on serum from each of the 51 participants. A doublet of 39/41 kDa was observed with serum from every participant except the null source patient, for whom APOL1 was undetectable (Fig. 1C and supplementary Fig. S2). While we cannot rule out a gene-dosage effect on expression, we did not observe any consistent decrease in band intensity from heterozygous patients compared to wild type controls. Urine samples were also examined for the presence of APOL1 by immunoblot, but even after concentrating urine 50X by lyophilization and using dilute serum as a weak positive control, APOL1 was undetectable in urine samples (supplementary Fig. S3).

**Figure 2:**
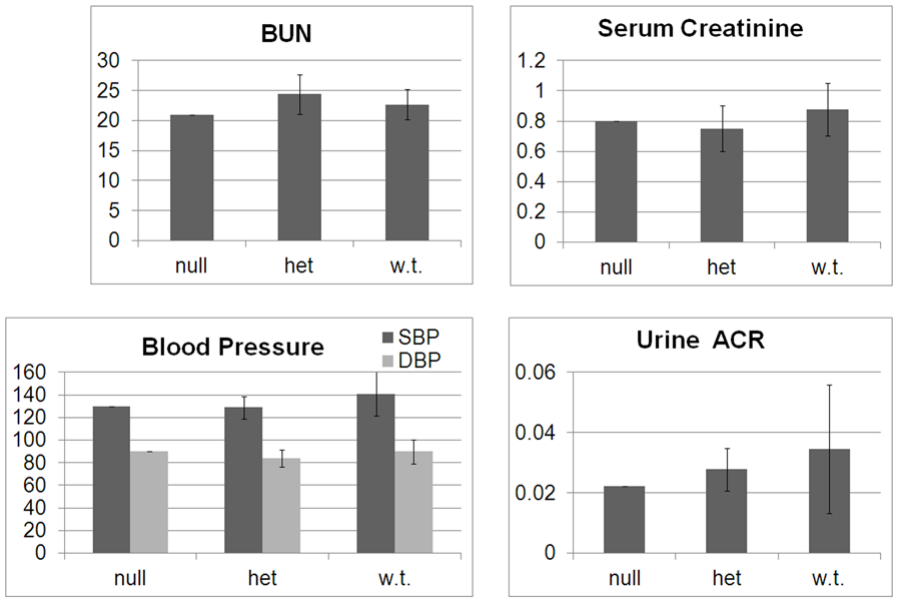
Clinical Data. From 51 subjects, clinical data for blood pressure, serum BUN, serum creatinine, and urine albumin∶creatinine ratios were sorted by genotype for comparison: “null” = homozygous null (N = 1); “het” = heterozygote (N = 8); “w.t.” = wild type (N = 42). Error bars represent S.D.

From the Past Medical History and Family History, there were very few clinical suggestions of kidney disease among the 51 participants: one female participant had a single episode of nephrolithiasis, the brother of the source patient had sudden death of unknown cause at the age of 42 (among the many possible causes of his death are kidney disease or death due to an infection similar to his brother's), and there were two instances of sudden death at a young age of unclear cause; in both cases the report of preexisting fever and rash by the mother suggests one of the mosquito-borne viruses endemic to the area. Nothing else in the past medical history or family history of any participants was suggestive of kidney disease. Clinical data obtained from all 51 participants included blood pressure, blood urea nitrogen, serum creatinine, and urine albumin∶urine creatinine ratio. Based on both Sanger sequencing and immunoblotting, we confirmed the previously identified trans-heterozygous null source patient, and we identified 8 new people heterozygous for either allele A or allele B. We found no evidence of a genotype-phenotype correlation; there was no difference in any of the clinical parameters between the *APOL1* null source patient, the 8 individuals who are heterozygous for either null allele, and the 42 individuals who are wild-type for each of these alleles (Fig. 2). In particular, the *APOL1* null source patient, with the exception of likely osteoporosis of the hip, has recovered from his critical illness due to *T. evansi* and has no evidence of glomerulosclerosis based on blood pressure, blood urea nitrogen, serum creatinine, or albuminuria (Fig. 2, “null”).

**Figure 3:**
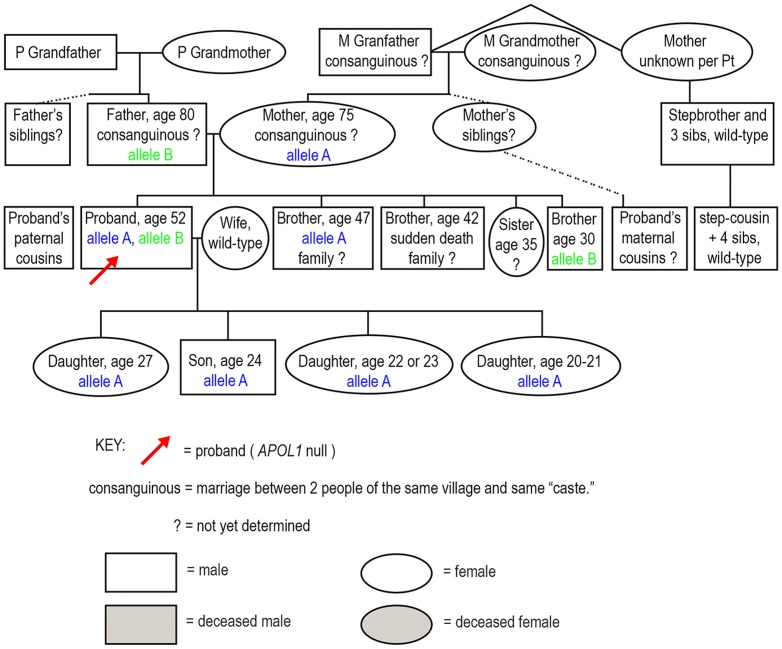
The inheritance of alleles A and B are depicted within a family dendogram, which also illustrates salient clinical findings. Other study participants not depicted in this dendogram were wild type for both *APOL1* null alleles and were not part of the immediate family.

Based on the family history, genotyping and immunoblotting, a family tree was constructed to illustrate the distribution of alleles and the potential for finding additional null persons (Fig. 3). At this time we have not identified any new null individuals but we are continuing to search for additional members of the extended family.

## Discussion

The history of the locus at Chr22 that contributes to African-American focal and segmental glomerulosclerosis (FSGS) and HIV-associated nephropathy (HIVAN) is a fascinating story that illustrates the strengths and weaknesses of genome wide association analysis. As described in the Introduction, the initial assumption that *MYH9* was the causal disease gene was based on both the high genome wide correlation of polymorphisms, and because rare, autosomal dominant *MYH9* mutations in patients with Epstein's and Fetchner's syndrome result in a glomerular disease that is histologically similar to idiopathic FSGS [2], [3], [46]. *MYH9* was subsequently supplanted as the candidate disease gene when new data revealed that SNPs within *APOL1* correlate with African-American FSGS even more robustly and can account for most or all of the effect previously attributed to SNPs within *MYH9*. Current confidence that *APOL1* is the correct disease gene is, once again, extremely high. However, as reviewed in the Introduction, while the evidence that the G1 and G2 variants of APOL1 cause increased resistance to SRA-expressing Trypanosomes is strong, and increased parasite resistance provides a logical explanation for the high allele frequency of G1 and G2 alleles in African-American and sub-Saharan populations, there are no data demonstrating the causality of *APOL1* alleles in glomerulosclerosis. Unlike the intronic SNPs first identified within *MYH9*, the G1 and G2 alleles of *APOL1* are coding polymorphisms (a missense and an in-frame deletion, respectively), and while this may initially appear to support causation, the caveat of linkage from GWAS discoveries applies similarly to all polymorphisms, be they in exons, introns, or intergenic regions.

Previous investigators have raised the possibility that the predisposition to glomerulosclerosis among African-Americans related to the Chr22 locus has not been fully explained [11], [30], [39]. Based on our findings we propose that African-American glomerulosclerosis results, not from the G1 and G2 variants themselves, but from “genetic hitchhiking” of deleterious mutations along with the G1 and G2 alleles. The basic principle of this hypothesis, that a phenotype initially ascribed to one mutation may instead result from another, linked mutation, is encountered in random mutagenesis screens in *Saccharomyces*, *Drosophila*, *Arabadopsis* and *C. elegans* [47], [48]. In human genetics, linkage is also a caveat of genome wide association [18], but perhaps been less of a concern as very few candidate disease genes have been misidentified initially based on linkage (a notable exception being the current interpretation of *MYH9* “risk haplotypes”). However, this phenomenon has recently been examined in greater detail among human populations, and the term “genetic hitchhiking” has been coined [44]. One recent example for which genetic hitchhiking has been proposed involves allele 503F of the ergothionene transporter *OCTN1*. Allele 503F correlates with an increased risk for Crohn's disease, but causal proof and a mechanism by which *OCTN1* contributes to gastrointestinal autoimmunity in Crohn's disease has been elusive. Hypotheses have included molecular mimicry between a gastrointestinal pathogen and the *OCTN*1 polymorphism, or a change in gastrointestinal proclivity to infection as a result of the transporter polymorphism, none of which have gained widespread support. The recent alternative hypothesis of genetic hitchhiking proposes that *OCTN1* 503F has no direct role with Crohn's per se, but rather improves uptake of ergothionene, which was a limiting nutrient in cultivated crops at the time of the Fertile Crescent. As the 503F allele was positively selected during the process of human migration out of the Fertile Crescent, other sequence variants in IRF-1 that are linked to 503F and cause Crohn's disease “hitchhiked” along during a selective sweep [45]. While the *a priori* candidate was nearby *IL5* (interleukin-5), the investigators instead found that *IRF-1*, which encodes interferon regulatory factor-1 and is also linked to *OCTN1*, appears to be the culprit. In a second example of “genetic hitchhiking,” the SLC24A5 allele was positively selected in Europeans for the effect of decreasing freckles, but this allele also caries several rare, linked, deleterious mutations in *FBN1*, several of which have been shown to cause Marfan's syndrome [44]. None of these *FBN1* polymorphisms is sufficiently common to rise to genome wide significance in moderately sized studies, but when several of the polymorphisms are all linked to the reduced freckling phenotype of SLC24A5, then lack of freckles can correlate with Marfan's syndrome.

**Figure 4:**
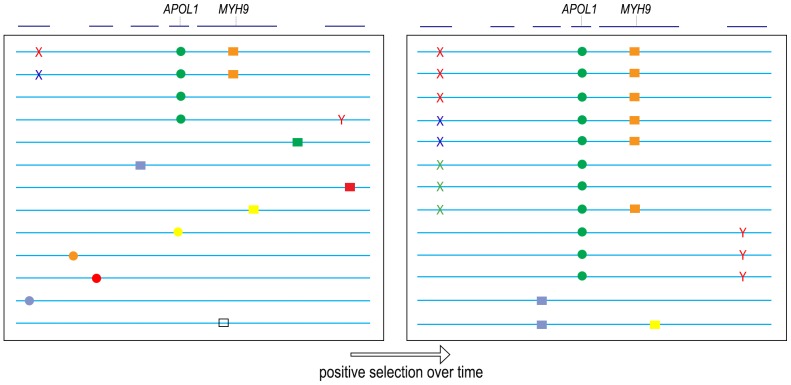
Model of “Genetic Hitchhiking” at the *MYH9*/*APOL1* locus. All genes at the locus (blue lines, top) are not depicted. The G1 and G2 *APOL1* polymorphisms (green circle) undergo positive selection over time due to increased resistance against Trypanosoma, eventually reaching high allele frequency. In the process, other nearby polymorphisms “hitchhike” along with G1 and G2, including intronic polymorphisms in *MYH9* (orange box), and hypothesized “glomerulosclerosis polymorphisms” in linked disease genes that have not been identified. The number of such “glomerulosclerosis polymorphisms” and the number of genes involved (1 or several) is not known. For the purposes of this cartoon we depict three hitchhiking polymorphisms (Xs of different colors) in a disease gene on one side of *APOL1*, and 1 polymorphism (Y) in a second disease gene on the other side of *APOL1*. We hypothesize that each individual “glomerulosclerosis polymorphism” is present at low frequency and has escaped statistical significance within the cohorts that have been examined, but in aggregate, multiple “glomerulosclerosis polymorphisms” that hitchhiked along with *APOL1* may account fully for disease. Possible methods of identifying such glomerulosclerosis polymorphisms are proposed in the Discussion. This hypothesis of multiple, rare polymorphisms that hitchhike along with G1 and G2 could explain why *APOL1* correlates highly with glomerulosclerosis and yet only ∼5% of G1/G2 homozygous individuals develop glomerulosclerosis (those 5% of G1/G2 persons who are also homozygous for the hitchhiking “glomerulosclerosis polymorphisms”). Lastly, we make no assumptions about the evolution of these causal “glomerulosclerosis polymorphisms,” which may have been present in *cis* to G1/G2 before a selective sweep (red and blue X, red Y), or may have arisen during or late in the process of positive selection for G1/G2 (green X). Two recombination events are depicted, and while neither is informative, recombination breakpoints of uncommon HIVAN or FSGS patients who do not have 2 alleles of G1/G2 may be highly informative (see Discussion).

A similar phenomenon of genetic hitchhiking is consistent with the data for the G1 and G2 alleles of *APOL1* (Fig. 4). We hypothesize that the reproductive advantage of resistance to infection by Trypanosoma and possibly other parasites led to a selective sweep of the G1 and G2 alleles in recent evolutionary history, and during this selective sweep additional sequence variants co-segregated with the G1 and G2 alleles, some of which are deleterious “glomerulosclerosis polymorphisms.” This hypothesis is consistent with the G1 and G2 alleles demonstrating the most mathematically robust correlation with FSGS, because they are the site of positive selection and must be present on every haplotype, whereas multiple “glomerulosclerosis polymorphisms” that have hitchhiked along with G1/G2 over the course of positive selection will each be present at a lower allele frequency and would elicit only a small signal in comparison with G1/G2. Moreover, the lower frequency of these “glomerulosclerosis polymorphisms” might explain why only 5% of G1/G2 homozygotes develop FSGS.

The strength of our study is that it provides one instance of an inconsistency with the prevailing theory for *APOL1*: if the G1 and G2 alleles predispose to glomerulosclerosis in a recessive model, suggesting a loss of function mutation, then a gentleman with a canonical loss of function mutation (a null allele) should have at least as high a predilection for kidney disease. In model organism classical genetics, a canonical null mutation often results in a stronger phenotype than missense loss of function mutations, but this is not universally true. Accordingly, we must make an assumption about the hypothetical penetrance of FSGS in an *APOL1* null individual if a loss of function does cause disease. For the sake of argument, assuming a higher phenotypic penetrance of 50% glomerulosclerosis with null alleles, then we would require the absence of glomerulosclerosis in 6 *APOL1* null people to show significance by Chi-square analysis (two tailed p = 0.0455). Based on these estimates, our study is underpowered for more definitive conclusions and to address this we have proposed extending our study to locate additional *APOL1* null individuals within the source patient's extended family.

The weakness of our hypothesis is that we have no data on which polymorphisms linked to *APOL1* are the best candidates for “genetic hitchhiking.” We also acknowledge that our study does not rule out all current models in which *APOL1* causes FSGS in concert with a gene-environment interaction, such as a specific viral co-infection, and it is possible that this unknown virus or environmental effect is not endemic to Eastern Maharashtra, India. That said, it is difficult to disprove a gene-environment viral interaction with the G1/G2 alleles when there is no data yet to support this as a direct mechanism. Third, while the phenotype of *APOL1*-associated glomerulosclerosis is usually described according to the incidence of end-stage renal disease (ESRD), for which G1 and G2 behave as recessive alleles as described in the introduction, there are other ways to classify the glomerulosclerosis phenotype. Among African-Americans with non-diabetic causes of ESRD, two groups recently reported that *APOL1* variants are associated with a younger age of dialysis initiation. One group found a younger age of dialysis initiation associated with the G1 allele in semi-dominant fashion [49], while a second group found a younger age of dialysis initiation association of G1 in recessive fashion but with a trend towards significance among heterozygotes [50]. In these studies, the G2 allele did not demonstrate a semi-dominant effect on age of dialysis initiation (and in the second study G2 showed no significant effect on age of dialysis initiation). While the details are complex and will require larger numbers in additional study, it is not unheard of for mutations in model organisms to behave as recessive alleles for one phenotype and dominant alleles for another phenotype. The differences between G1 and G2 within separate cohorts of patients and with distinct methods of phenotyping (incidence of glomerulosclerosis versus age of dialysis initiation) speaks to the genetic complexity at this locus, and if the dominance of G1 and G2 is subtle but real, then our finding of a lack of association between *APOL1* null alleles and glomerulosclerosis would be less surprising.

In the end, we suggest that assuming the G1 and G2 alleles are causal for FSGS has important policy ramifications and has important meaning to patients and advocacy groups. Prior to investing significant effort into screening asymptomatic individuals for *APOL1* G1 and G2 alleles, and looking for gene-environment interactions with *APOL1*, we suggest that additional effort focus on definitively establishing causation of glomerulosclerosis. In order to test for evidence of genetic hitchhiking, for instance, a few assumptions can be made: first, the allele frequency of each of these “glomerulosclerosis polymorophisms” must be low enough that the signal for any one variant has not risen to the level of genome wide significance in studies thus far. Second, because the risk of incident disease appears equal for homozygous G1/G1, G2/G2 and trans-G1/G2, these hypothesized “glomerulosclerosis polymorphisms” must fail to complement: this could arise from multiple “glomerulosclerosis polymorphisms” within the same gene, or multiple polymorphisms in different genes if the genes are epistatically related, as is seen for oligogenic inheritance with the Bardet-Biedl Syndrome and Nephronopthisis [51], [52]. To test this, multiple polymorphisms from a candidate gene could be collapsed into a single “bin” to look for a significant correlation with disease, or in the case of two genes linked to *APOL1* suspected of forming an epistatic group, polymorphisms from both genes would be collapsed into a single “bin.” A second approach, similar to the work on Crohn's disease, would be to examine differences in gene expression from kidney biopsies with or without FSGS, stratified by 0, 1, or 2 copies of the G1/G2 alleles, paying particular attention to genes within the several centimorgans on either side of APOL1 that constitutes the proposed range for “genetic hitchhiking.” [45]. Third, if other *APOL1* alleles from different human populations correlate significantly with glomerulosclerosis, this would support a causal role for *APOL1*, while a lack of correlation between additional *APOL1* variant alleles and kidney disease would favor an alternative hypothesis such as genetic hitchhiking. Fourth, in model organism genetics, a rare recombinant can provide key information of a gene or mechanism, and similar results might be obtained from G1 or G2 heterozygotes who still develop glomerulosclerosis, possibly through a recombination event in which the 2^nd^ copy of G1 or G2 is lost, but the presumed disease gene is retained at or after the site of recombination. Our hope is that these and other additional efforts will firmly establish the causal gene and provide a targetable mechanism to affected patients and to those asymptomatic individuals of African descent who carry the G1/G2 alleles.

## Materials and Methods

### Study Design

All clinical investigations were conducted according to the principles expressed in the Declaration of Helsinki. Proposals were reviewed approved by the Institutional Ethics Committee at Government Medical Hospital, Nagpur, Maharashtra State, India, and the Institutional Review Board at the University of Pennsylvania School of Medicine. Permissions were obtained from state, local and village authorities of Maharashtra State, including members of the police and the elected village leader of Seoni. The study was also discussed with both the WHO and the Indian Council of Medical Research. Informed consent from all participants was obtained with verbal and written descriptions in Marathi, the native language of this region of India. Exclusion criteria were age under 18 or the inability to given verbal and written consent independently. After enrollment, participants were de-identified with an alpha-numeric designation for use on all documents and biological samples. Physicians of the Public Health Office of Maharashtra who speak Marathi obtained the Past Medical History, the Family History, and measured blood pressure for all participants (instructional guidelines per JNC7). Blood pressures were higher than expected in many cases, but despite efforts to provide a calm environment and allow the participants to relax by relating their family history prior to obtaining sphygmomanometry, it was difficult to fully overcome the likelihood of a “white coat” effect on blood pressure measurements: the arrival of an entire team of physicians, including the 1^st^ or 2^nd^ sighting of a Caucasian, was a social event for the village and the excitement level was high. Blood was collected in SST tubes (BD medical, 367977) and K_2_EDTA tubes (BD medical 367861) labeled alpha-numerically and kept at 4°C until processing. Nurses instructed participants in Marathi on providing clean catch urine samples, which were labeled alpha-numerically and kept at 4°C until processing. All participants received food, refreshment, and compensation for their time and effort.

Biological samples were transported at 4°C by jeep from Seoni to Government Medical Hospital (GMH), Nagpur for initial processing. Blood in serum separator tubes was centrifuged at 2000 RPM (est. 1300–1500×g) for 10 minutes in a tabletop centrifuge and then aliquoted for subsequent testing and storage. Estimation of serum BUN was performed by the kinetic glutamate dehydrogenase enzymatic method and estimation of serum creatinine was performed by kinetic enzymatic method in an autoanalyser(XL-300) at GMH and the remaining serum was transported at 4°C to Chandigarh for additional analysis. Urine albumin∶creatinine ratios were determined by immunoturbidometry at the Institute of Clinical Endocriniology, Nagpur. Urine was centrifuged at 2000 RPM in a tabletop centrifuge and the supernatant was aliquoted for transport to Chandigarh. A subset of five samples underwent confirmatory urine albumin∶creatinine assays at LalPathLabs in Chandigargh. Blood in K_2_EDTA tubes was processed to obtain white cell pellets for subsequent genomic DNA preparation using a protocol that combined input from Dr. D. Magen and Dr. S. Thompson: 3–4 mL of blood in EDTA tubes were transferred to 15 mL sterile Falcon tubes, then RBC lysis buffer (Tris pH 7.6 10 mM, NaCl 10 mM, MgCl_2_ 5 mM, mixed from stock solutions into 2 liters of Bisleri™ drinking water given the unavailability of ddH_2_O) was added to a final volume of 15 mls. Tubes were gently mixed, incubated 10 min, then centrifuged by tabletop at 2000 RPM. Supernatants were disposed in bleach while pellets were resuspended in 15 mls RBC lysis buffer, incubated 10 minutes, and centrifuged a second time at 2000 RPM. After disposal of supernatants, white cell pellets were inverted to dry, then kept at 4°C for transport to Chandigarh.

### DNA Sequencing

In Chandigarh at the CSIR- Institute for Microbial Technology, white cell pellets were resuspended in SE buffer (NaCl 75 mM, EDTA 25 mM pH 8) to which was added 0.2 mL of 10% SDS and 0.5 mL of ProteinaseK 1 mg/mL. After incubation at 55°C×3 hrs, 1 mL of 5 M NaCl was added, followed by chloroform extraction (up to 3X), then isopropanol precipitation, then manual transfer of chromosomal DNA precipitates using pipette tips into a fresh Eppendorf containing 70% EtOH/30% TE buffer (Tris 10 mM, EDTA 1 mM pH 8) for desalting, after which pellets were allowed to dry, then resuspended in TE buffer. Genomic DNA yield and 260/280 ratios were measured by Nanodrop (Thermofisher). PCR reactions of 50 uL each were made from a mastermix of Complete II buffer (10X, Bioron), dNTPs (final 200 uM, NEngBiolabs), primers (final 0.2 uM each; left TTGTGCAGGAATGAGGCAGA and right CCCCTGTAAGCTTCTTTCTTGTGCT), TAQ polymerase (final 2 U, Biobasic) and either water (control) or genomic DNA (100 ng). One fifth of each PCR reaction (10 uL) was examined by gel electrophoresis and photographed by handheld Nikon Coolpix, while the remaining 4/5^th^ of each PCR reaction was purified, then quantified by Nanodrop, and then sequenced with ABI BigDye v3.1 Cycle Sequencing per ABI protocol, with 10–20 ng of purified PCR product template and 4 pmol primer (left nested GGCAGATGAGCTCCGTAAAGC, right nested CCACCTGTTCACCGCTTTCA, fwd internal ATGGAGTTGGGAATCACAGC, rev internal GCTGTGATTCCCAACTCCATC), followed by EtOH precipitation, resuspension of pellets in 10–12 uL of HiDi formamide, then loading for sequence analysis. Tracings were viewed with FinchTV v1.4. Backups of genomic DNA were prepared using FTA-elute cards (Whatman WB 120412) from droplets of blood in EDTA tubes and processed per manufacturer's instructions.

### Immunoblotting

In Chandigarh, serum samples were examined for APOL1 by immunoblot. Serum was diluted at 1/5 dilution with autoclaved water and protein content was quantified by Bradford assay reagent (Pierce 23238). Equal amounts of protein were loaded in each well and were separated by 12% SDS-PAGE, transferred to PVDF membranes, blocked with 5% dry milk, then immunblotted with 1∶3000 of α-APOL1 (Sigma HPA018885) and developed with ECL (Pierce 32132). Membranes were subsequently blotted with α-human serum albumin (Abcam, ab10241) at 1∶10000 dilution for loading controls and then photographed by Nikon Coolpix. Urine samples were assayed for APOL1 by concentrating 3 mls of urine by lyophilization, resuspending samples in loading buffer, separating by 12% SDS-PAGE and immunoblotting as above.

## Supporting Information

Figure S1 **Additional tracings as visualized with ABI Sequence Scanner.** As shown, when either allele A or B is visible there is a sharp transition from a clearly readable sequence to a signal of two nucleotides mixed together at roughly 50% signal strength each- one nucleotide is the wild type sequence, and the other nucleotide is as expected if the wild type sequence is offset by one nucleotide (allele A) or two nucleotides (allele B).
(TIF)

Figure S2 **Immunoblots were performed on serum from all 51 participants using the same methodology as for** **Figure 1C** **, but in all cases, APOL1 protein was readily detectable.** Numbers 101–151 represent the de-identified patients
(TIF)

Figure S3 **Immunoblots for APOL1 were performed on urine, both undiluted (not shown) and after concentration of 3 mls of urine by lyophilization as shown in this blot.** As a positive control, serum from an APOL1 wild type individual (#117) was diluted 5X to decrease signal intensity. While APOL1 may or may not be filtered at the glomerulus, it was not readily detectable in urine with or without concentration by lyophilization.
(TIF)

Table S1 **Summary sheet of sequencing findings for alleles A and B from all tracings.**
(DOCX)
