# Supplementary figures and images for: APOL1 Null Alleles from a Rural Village in India Do Not Correlate with Glomerulosclerosis
Source: PLoS One. 2012 Dec 26;7(12):e51546. doi: 10.1371/journal.pone.0051546 (PMC3530541; doi:10.1371/journal.pone.0051546)

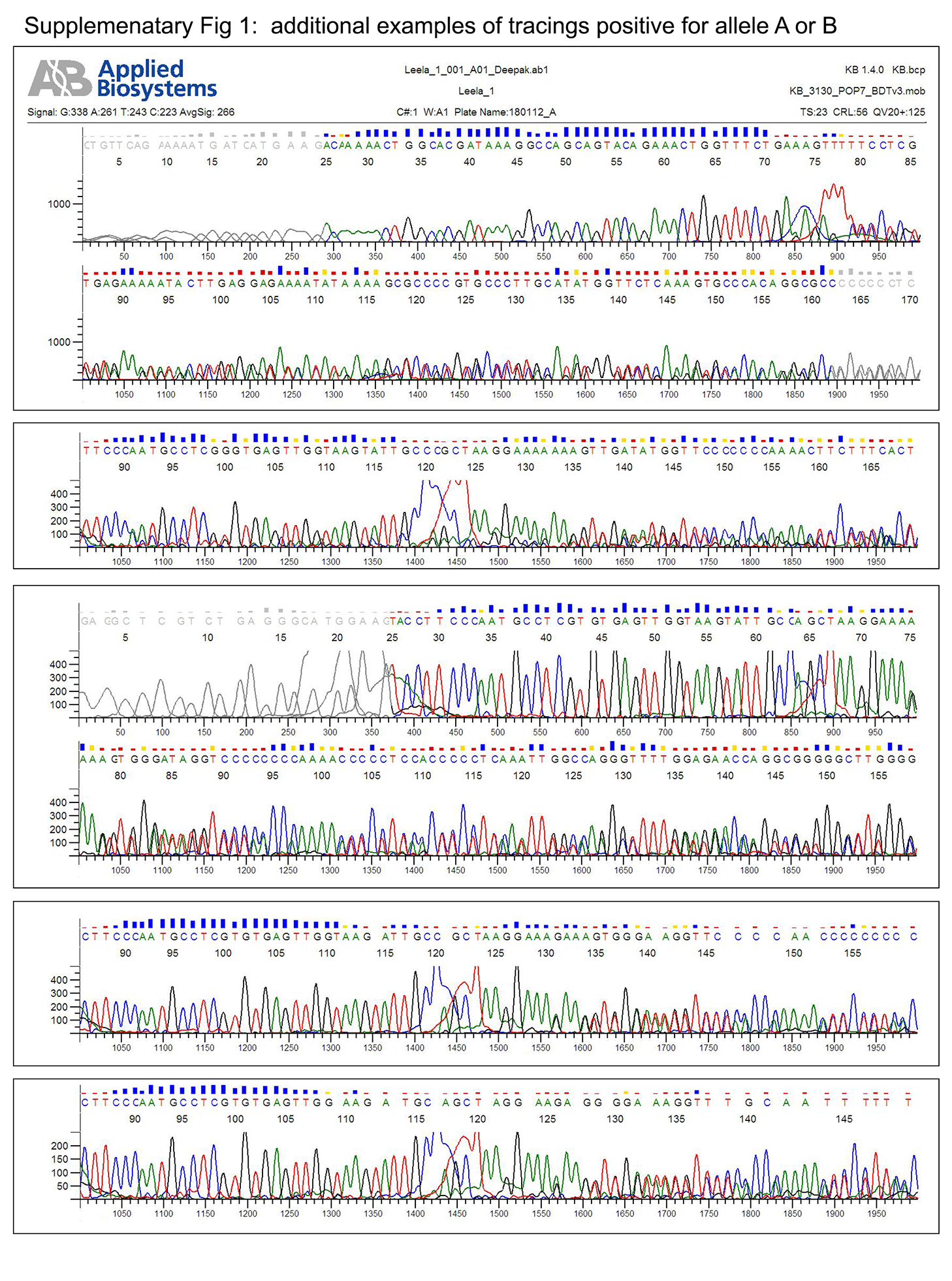

Supplement: Figure S1 — Additional tracings as visualized with ABI Sequence Scanner. As shown, when either allele A or B is visible there is a sharp transition from a clearly readable sequence to a signal of two nucleotides mixed together at roughly 50% signal strength each- one nucleotide is the wild type sequence, and the other nucleotide is as expected if the wild type sequence is offset by one nucleotide (allele A) or two nucleotides (allele B). (TIF) [file pone.0051546.s001.tif]

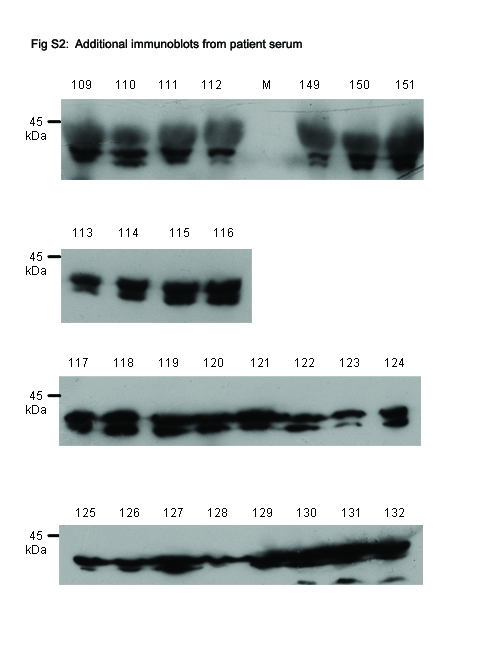

Supplement: Figure S2 — Immunoblots were performed on serum from all 51 participants using the same methodology as for Figure 1C , but in all cases, APOL1 protein was readily detectable. Numbers 101–151 represent the de-identified patients (TIF) [file pone.0051546.s002.tif]

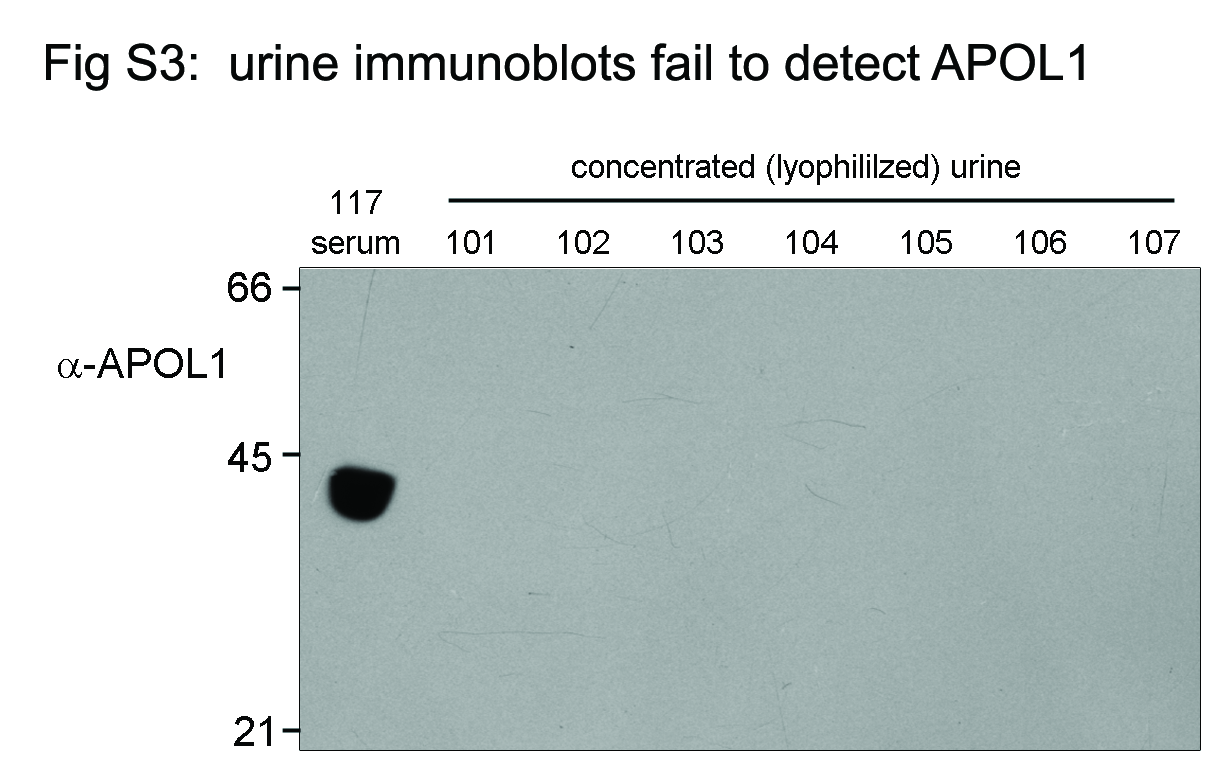

Supplement: Figure S3 — Immunoblots for APOL1 were performed on urine, both undiluted (not shown) and after concentration of 3 mls of urine by lyophilization as shown in this blot. As a positive control, serum from an APOL1 wild type individual (#117) was diluted 5X to decrease signal intensity. While APOL1 may or may not be filtered at the glomerulus, it was not readily detectable in urine with or without concentration by lyophilization. (TIF) [file pone.0051546.s003.tif]
