# Supplementary material for: APOL1 Null Alleles from a Rural Village in India Do Not Correlate with Glomerulosclerosis
Source: PLoS One. 2012 Dec 26;7(12):e51546. doi: 10.1371/journal.pone.0051546 (PMC3530541; doi:10.1371/journal.pone.0051546)
Supplement: Table S1 — Summary sheet of sequencing findings for alleles A and B from all tracings. (DOCX) [file pone.0051546.s004.docx]

Supplementary Table 1:

ABI BigDye v3.1 Cycle Sequencing. Summary of Tracings

| 15/1/12 | genomic DNA | primer | Lane on Seq Gel | Findings |
| --- | --- | --- | --- | --- |
| 1 | 101 | Pink left | 18/1/12: 1A | allele B is present, but read is dirty. CTGA before deletion, then mixed bases but with an unexplained C peak bridging three bases, followed by 3 unusually high T peaks, and later a G peak that comes between two base calls. Probably right, but will repeat. |
| 2 | 103 | Pink left | 15/1/12: G5 | wild type for allele B  But, allele A is present  with deletion of C with mixed read of bases afterwards at least through  **TTTCCTTAGCTGGCA** |
| ~~3~~ | ~~104~~ | ~~Pink left~~ | - | - repeated below |
| 4 | 105 | Pink left | 18/1/12: 1B | allele B probably present, but like 101 above, there is an artifactual very large C signal, followed by an artifactual large T signal just overlying the mutation site. Will repeat |
| 5 | 108 | Yellow Right | 15/1/12: H5 | wild type for allele A  But, allele B is present at end of red, with deletion of AA and mixed bases afterwards, at least through  **CAGAAA**  **CTGGTTTCTGA** |
| 6 | 101 | Yellow Right | 15/1/12: A6 | allele A is present  deletion of C with mixed read of bases afterwards, at least through  GGGACCTATCCCACTTTT |
| 7 | 103 | Yellow Right | 15/1/12: B6 | allele A is present  deletion of C with mixed read of bases afterwards to  TATCAACTTTT |
| 8 | 104 | Yellow Right | 15/1/12: C6 | allele A is present  deletion of C with mixed read after, at least through  **TTTTT**  **GGGTGAGAACATATCCAACTTT** |
| 9 | 105 | Yellow Right | 15/1/12: D6 | wild type for allele A site  But allele B is present at end of sequence (good read), with mixed bases after to **AACTGGTTTCTGA** |
| 10 | 107 | Yellow Right | 15/1/12: E6 | Not excellent sequence, but allele A is present, with mixed read of bases afterwards at least through  **AGGAGGTGAGGGAGTTTTT**  **GGGTGAGAACATATCCAACTTT** |
|  |  |  |  |  |

| 16/1/12  sample# | genomic DNA | primer | Lane on Seq Gel | Findings |
| --- | --- | --- | --- | --- |
| 1 | 104 | Pink left | 18/1/12: 1C | Allele B probably NOT present, as there is no shift in base calls downstream, but like 101 above ,the mutation site is covered by a large artifactual C and T peak.  Allele A is present at end of read, clean enough to read well, with mixed base calls afterwards at least through **TTTCCTTAGCTGGCA** |
| 2 | 107 | Pink left | 18/1/12: 1D | Allele B probably NOT present, as there is no shift in base calls downstream, but like 101 above ,the mutation site is covered by a large artifactual C and T peak.  Allele A is present at end of read, clean enough to read well, with mixed base calls afterwards at least through **TTTCCTTAGCTGGCA** |
| 3 | 108 | Pink left | 18/1/12: 1E | Likely, allele B is present, based on mixed base calls downstream, but very heavy artifactual C and T peak obscures mutation site. |
| 4 | 109 | Pink left | 18/1/12: 1F | Allele B probably NOT present, as there is no shift in base calls downstream, but like 101 above ,the mutation site is covered by a large artifactual C and T peak.  Wild type for allele A at end of read, clean enough to call well. |
| 5 | 110 | Pink left | 18/1/12: 1G | Allele B probably NOT present, as there is no shift in base calls downstream, but like 101 above ,the mutation site is covered by a large artifactual C and T peak  Allele A is present at end of read, clean enough to read well, with mixed base calls afterwards at least through **TTTCCTTAGCTGGCA** |
| 6 | 112 | Pink left | 18/1/12: 1H | Allele B probably NOT present, as there is no shift in base calls downstream, but like 101 above ,the mutation site is covered by a large artifactual C and T peak.  Wild type for allele A at end of read, clean enough to call well. |
| 7 | 113 | Pink left | 18/1/12: 2A | Allele B probably NOT present, as there is no shift in base calls downstream, but like 101 above ,the mutation site is covered by a large artifactual C and T peak.  Allele A is present at end of read, clean enough to read well, with mixed base calls afterwards at least through **TTTCCTTAGCTGGCA** |
|  |  |  |  |  |

| 16/1/12  sample# | genomic DNA | primer | Lane on Seq Gel | Findings |
| --- | --- | --- | --- | --- |
| 1 | 103 | Yellow Right (repeat) | 18/1/12: 2B | Allele A is present. The sequence has a large C,T,G artifact, ugly, just before the site, but mutation is fairly clean, and downstream, there are clean mixed base reads at least through **GGTGAGAACATATCCAACTTT** |
| 2 | 104 | Yellow Right  (repeat) | 18/1/12: 2C | Allele A is present. The sequence has a large C,T,G artifact, ugly, just before the site, but mutation is fairly clean, and downstream, there are clean mixed base reads at least through **GGTGAGAACATATCCAACTTT** |
| 3 | 107 | Yellow Right (repeat) | 18/1/12: 2D | Allele A is present. The sequence has a large C,T,G artifact, ugly, just before the site, but mutation is fairly clean, and downstream, there are clean mixed base reads at least through **GGTGAGAACATATCCAACTTT** |
| 4 | 108 | Yellow Right | 18/1/12: 2E | Wild type for allele A.  Allele B is present at end of read, with mixed base calls at least through **ACTGGTTTCTGA** |
| 5 | 109 | Yellow Right | 18/1/12: 2F | Wild type for allele A. The sequence has a large C,T,G artifact, ugly, just before the site, but the TTTCTTTC is clean.  Wild type for allele B. |
| 6 | 110 | Yellow Right | 18/1/12: 2G | Allele A is present. The sequence has a large C,T,G artifact, ugly, just before the site, but mutation is fairly clean, and downstream, there are clean mixed base reads at least through **GGTGAGAACATATCCAACTTT** |
| 7 | 112 | Yellow Right | 18/1/12: 2H | wild type for allele A (with large CTG artifact just before the site).  wild type for allele B |
| 8 | 113 | Yellow Right | 18/1/12: 3A | Allele A is present. The sequence has a large C,T,G artifact, ugly, just before the site, but mutation is fairly clean, and downstream, there are clean mixed base reads at least through **TTTTGGGTGAGAACATATCCAACTTT** |
|  |  |  |  |  |

| 17/1/12 | genomic DNA | primer | Lane on Seq Gel | Findings |
| --- | --- | --- | --- | --- |
| Y 114 | 114 | Yellow Right | 18/1/12: 3B | wild type for allele A, and wild type for allele B at the end of the read. Clean base calls. |
| Y 115 | 115 | Yellow Right | 18/1/12: 3C | wild type for allele A, and wild type for allele B at the end of the read. Clean base calls. |
| Y 117 | 117 | Yellow Right | 18/1/12: 3D | wild type for allele A, and wild type for allele B at the end of the read. Clean base calls. |
| Y 118 | 118 | Yellow Right | 18/1/12: 3E | wild type for allele A, and wild type for allele B at the end of the read. Clean base calls. |
| Y 120 | 120 | Yellow Right | 18/1/12: 3F | wild type for allele A, and wild type for allele B at the end of the read. Clean base calls. |
| Y 122 | 122 | Yellow Right | 18/1/12: 3G | wild type for allele A, and wild type for allele B at the end of the read. Clean base calls. |
| Y 123 | 123 | Yellow Right | 18/1/12: 3H | wild type for allele A, and wild type for allele B at the end of the read. Clean base calls. |
| Y 124 | 124 | Yellow Right | 18/1/12: 4A | wild type for allele A, and wild type for allele B at the end of the read. Clean base calls. |
| Y 125 | 125 | Yellow Right | 18/1/12: 4B | wild type for allele A, and wild type for allele B at the end of the read. Clean base calls. |
| Y 127 | 127 | Yellow Right | 18/1/12: 4C | wild type for allele A, and wild type for allele B at the end of the read. Clean base calls. |
| Y 128 | 128 | Yellow Right | 18/1/12: 4D | wild type for allele A, and wild type for allele B at the end of the read. Clean base calls. |
| Y 130 | 130 | Yellow Right | 18/1/12: 4E | wild type for allele A, and wild type for allele B at the end of the read. Clean base calls. |
| Y 132 | 132 | Yellow Right | 18/1/12: 4F | wild type for allele A, and wild type for allele B at the end of the read. Clean base calls. |
| Y 133 | 133 | Yellow Right | 18/1/12: 4G | wild type for allele A, and wild type for allele B at the end of the read. Clean base calls. |
| Y 134 | 134 | Yellow Right | 18/1/12: 4H | wild type for allele A, and wild type for allele B at the end of the read. Clean base calls. |
| Y 135 | 135 | Yellow Right | 18/1/12: 5A | wild type for allele A, and wild type for allele B at the end of the read. Clean base calls. |
| Y 136 | 136 | Yellow Right | 18/1/12: 5B | wild type for allele A, and wild type for allele B at the end of the read. Clean base calls. |
| Y 137 | 137 | Yellow Right | 18/1/12: 5C | wild type for allele A, and wild type for allele B at the end of the read. Clean base calls. |
| Y 138 | 138 | Yellow Right | 18/1/12: 5D | wild type for allele A, and wild type for allele B at the end of the read. Clean base calls. |
| Y 139 | 139 | Yellow Right | 18/1/12: 5E | wild type for allele A, and wild type for allele B at the end of the read. Clean base calls. |
| Y 140 | 140 | Yellow Right | 18/1/12: 5F | wild type for allele A, and wild type for allele B at the end of the read. Clean base calls. |
| Y 141 | 141 | Yellow Right | 18/1/12: 5G | wild type for allele A, and wild type for allele B at the end of the read. Clean base calls. |
| Y 142 | 142 | Yellow Right | 18/1/12: 5H | wild type for allele A, and wild type for allele B at the end of the read. Clean base calls. |
| Y 144 | 144 | Yellow Right | 18/1/12: 6A | wild type for allele A, and wild type for allele B at the end of the read. Clean base calls. |
| Y 145 | 145 | Yellow Right | 18/1/12: 6B | wild type for allele A, and wild type for allele B at the end of the read. Clean base calls. |
| Y 146 | 146 | Yellow Right | 18/1/12: 6C | wild type for allele A, and wild type for allele B at the end of the read. Clean base calls. |
| Y 147 | 147 | Yellow Right | 18/1/12: 6D | wild type for allele A, and wild type for allele B at the end of the read. Clean base calls. |
| Y 148 | 148 | Yellow Right | 18/1/12: 6E | wild type for allele A, and wild type for allele B at the end of the read. Clean base calls. |
| Y 151 | 151 | Yellow Right | 18/1/12: 6F | wild type for allele A, and wild type for allele B at the end of the read. Clean base calls. |
|  |  |  |  |  |

| 17/1/12 | genomic DNA | primer | Lane on Seq Gel | Findings |
| --- | --- | --- | --- | --- |
| P 114 | 114 | Pink Left | 18/1/12: 6G | wild type for allele B, and wild type for allele A at the end of the read. Clean base calls. |
| P 115 | 115 | Pink Left | 18/1/12: 6H | wild type for allele B, and wild type for allele A at the end of the read. Clean base calls |
| P 117 | 117 | Pink Left | 18/1/12: 7A | wild type for allele B, and wild type for allele A at the end of the read. Clean base calls. |
| P 118 | 118 | Pink Left | 18/1/12: 7B | Probably wild type for allele B, but a large artifactual C.T is present around the site. Wild type for allele A at end of read with clean base calls. |
| P 120 | 120 | Pink Left | 18/1/12: 7C | Probably wild type for allele B, but a large artifactual C.T is present around the site. Wild type for allele A at end of read with clean base calls. |
| P 122 | 122 | Pink Left | 18/1/12: 7D | Probably wild type for allele B, but a large artifactual C.T is present around the site. Wild type for allele A at end of read with clean base calls. |
| P 123 | 123 | Pink Left | 18/1/12: 7E | wild type for allele B, and wild type for allele A at the end of the read. Fairly clean base calls. |
| P 124 | 124 | Pink Left | 18/1/12: 7F | wild type for allele B, and wild type for allele A at the end of the read. Fairly clean base calls. |
| P 125 | 125 | Pink Left | 18/1/12: 7G | wild type for allele B, and wild type for allele A at the end of the read. Clean base calls. |
| P 127 | 127 | Pink Left | 18/1/12: 7H | Probably wild type for allele B, but a large artifactual C.T is present around the site. Wild type for allele A at end of read with clean base calls. |
| P 128 | 128 | Pink Left | 18/1/12: 8A | Probably wild type for allele B, but a large artifactual C.T is present around the site. Wild type for allele A at end of read with clean base calls. |
| P 130 | 130 | Pink Left | 18/1/12: 8B | Probably wild type for allele B, but a large artifactual C.T is present around the site. Wild type for allele A at end of read with clean base calls. |
| P 132 | 132 | Pink Left | 18/1/12: 8C | wild type for allele B, and wild type for allele A at the end of the read. Fairly clean base calls. |
| P 133 | 133 | Pink Left | 18/1/12: 8D | Probably wild type for allele B, but a large artifactual C.T is present around the site. Wild type for allele A at end of read with clean base calls. |
| P 134 | 134 | Pink Left | 18/1/12: 8E | Probably wild type for allele B, but a large artifactual C.T is present around the site. Wild type for allele A at end of read with clean base calls. |
| P 135 | 135 | Pink Left | 18/1/12: 8F | wild type for allele B, and wild type for allele A at the end of the read. Fairly clean base calls. |
| P 136 | 136 | Pink Left | 18/1/12: 8G | wild type for allele B, and wild type for allele A at the end of the read. Clean base calls. |
| P 137 | 137 | Pink Left | 18/1/12: 8H | wild type for allele B, and wild type for allele A at the end of the read. Fairly clean base calls. |
| P 138 | 138 | Pink Left | 18/1/12: 9A | wild type for allele B, and wild type for allele A at the end of the read. Fairly clean base calls. |
| P 139 | 139 | Pink Left | 18/1/12: 9B | Probably wild type for allele B, but a large artifactual C.T is present around the site. Wild type for allele A at end of read with clean base calls. |
| P 140 | 140 | Pink Left | 18/1/12: 9C | Probably wild type for allele B, but a large artifactual C.T is present around the site. Wild type for allele A at end of read with clean base calls. |
| P 141 | 141 | Pink Left | 18/1/12: 9D | wild type for allele B, and wild type for allele A at the end of the read. Fairly clean base calls. |
| P 142 | 142 | Pink Left | 18/1/12: 9E | wild type for allele B, and wild type for allele A at the end of the read. Fairly clean base calls. |
| P 144 | 144 | Pink Left | 18/1/12: 9F | Probably wild type for allele B, but a large artifactual C.T is present around the site. Wild type for allele A at end of read with clean base calls. |
| P 145 | 145 | Pink Left | 18/1/12: 9G | Wild type for both allele A and allele B. Very clean reads. |
| P 146 | 146 | Pink Left | 18/1/12: 9H | Probably wild type for allele B, but a large artifactual C.T is present around the site. Wild type for allele A at end of read with clean base calls. |
| P 147 | 147 | Pink Left | 18/1/12: 10A | wild type for allele B, and wild type for allele A at the end of the read. Fairly clean base calls. |
| P 148 | 148 | Pink Left | 18/1/12: 10B | wild type for allele B, and wild type for allele A at the end of the read. Fairly clean base calls. |
| P 151 | 151 | Pink Left | 18/1/12: 10C | Probably wild type for allele B, but a large artifactual C.T is present around the site. Wild type for allele A at end of read with clean base calls. |
|  |  |  |  |  |

| 20/1/12 | Repeats | Primer | Lane on Seq gel | Findings |
| --- | --- | --- | --- | --- |
| 101p | 101 | Pink left | A1 | Much better. Clean read of allele B, mutation is present w mixed bases afterwards. Tiny artifact of C/T peak present. |
| 101.5 | 101 w ½ template | Pink left | B1 | Not as good as A1. Mutant allele B is present. Artifact of C peak is worse. |
| 104p | 104 | Pink left | C1 | Large artifact of C around allele B site. Very clean read of allele A, mutation is present w mixed read thereafter. |
| 104.5 | 104 w ½ template | Pink left | D1 | As above, C1. No difference w ½ template. What is this C/T artifact at same place in every run? |
| 107p | 107 | Pink left | E1 | Large artifact of C and T around allele B site, once again.  Very clean read of allele A, mutation is present w mixed read thereafter |
| 107.5 | 107 w ½ template | Pink left | F1 | As above, E1. No change w ½ template. |
| 108p | 108 | Pink left | G1 | Mutant allele B is present, but the artifact is still there, just like every above, a large C/T peak spanning 3-5 base calls. |
| 108.5 | 108 w ½ template | Pink left | H1 | Just like G1. No difference w lower template. There is some systematic error- why on exactly the same place in the run in every sample? Is this a problem w the gel apparatus itself? |

Repeats

Yogendra

| 06/02/12 | genomic DNA | primer | Lane on Seq Gel | Findings |
| --- | --- | --- | --- | --- |
| 1 | 102 | Yellow Right | 06/02/12: A5 | Wild type for allele A and B. Read is quite impressive and both alleles can be read. |
| 2 | 106 | Yellow Right | 06/02/12: B5 | allele A is present  with deletion of C with mixed read of bases afterwards at least through  **GGTGAGAACATATCCAACTTTC** |
| 3 | 111 | Yellow Right | 06/02/12: C5 | Wild type for allele A and B with good read. |
| 4 | 116 | Yellow Right | 06/02/12: D5 | Wild type for both the alleles A and B, but there is an unexplainable overlapping pick of A and G downstream the seq **TCGGTTGAAAAGT**, may be just an artifact.  Actually 3 equivocal bases are present, widely spaced, but each is likely artifact- wait to verify from other strand w pink primer. (And note same artifact in many lanes below…) |
| **5** | **119** | **Yellow Right** | **lost** | **redone- see below** |
| 6 | 121 | Yellow Right | 06/02/12: E5 | Wild type for both the alleles A and B, but there is an unexplainable overlapping pick of A and G downstream the seq **TCGGTTGAAAAGT**, may be just an artifact. 2 other artifacts shared w D5 read. |
| 7 | 126 | Yellow Right | 06/02/12: F5 | Wild type for allele A and B. Read is quite impressive and both alleles can be read. |
| 8 | 129 | Yellow Right | 06/02/12: G5 | Wild type for allele A and B with good read. |
| 9 | 131 | Yellow Right | 06/02/12: H5 | wild type for allele A site  but allele B cannot be read. |
| 10 | 143 | Yellow Right | 06/02/12: A6 | Wild type for both the alleles A and B, but there is an unexplainable overlapping pick of A and G downstream the seq **TCGGTTGAAAAGT**, may be just an artifact ( same group of 3 artifacts as D5) |
| 11 | 149 | Yellow Right | 06/02/12: B6 | Wild type for both the alleles A and B, but there is an unexplainable overlapping pick of A and G downstream the seq **TCGGTTGAAAAGT**, may be just an artifact (same group of 3 artifacts as D5). |
| 12 | 150 | Yellow Right | 06/02/12: C6 | Wild type for both the alleles A and B.  Artifact here is extremely tiny. |

| 14/02/12 | genomic DNA | primer | Lane on Seq Gel | Findings |
| --- | --- | --- | --- | --- |
|  | 119 | **Yellow right** | 14/02/12: G6 | Wild type for allele A and B. Read is quite impressive and both alleles can be read. |
|  | 102 | Pink left | 14/02/12: H6 | Wild type for allele A and B. Read is quite impressive and both alleles can be read. |
|  | 106 | Pink left | 14/02/12: A7 | Wild type for allele B  Allele “A” is present  w mixed, appropriate read at least through  **CTTTCCTTAGCTGGCAATACTT** |
|  | 111 | Pink left | 14/02/12: B7 | Wild type for allele A and B. Read is quite impressive and both alleles can be read. |
|  | 116 | Pink left | 14/02/12: C7 | Wild type for allele A and B. Read is quite impressive and both alleles can be read. |
|  | **119** | Pink left | 14/02/12: D7 | Wild type for allele A and B. Read is quite impressive and both alleles can be read. |
|  | 121 | Pink left | 14/02/12: E7 | Wild type for allele A and B. Read is quite impressive and both alleles can be read. |
|  | 126 | Pink left | 14/02/12: F7 | Wild type for allele A and B. Read is quite impressive and both alleles can be read. |
|  | 129 | Pink left | 14/02/12: G7 | Wild type for allele A and B. Read is quite impressive and both alleles can be read. |
|  | 131 | Pink left | 14/02/12: H7 | Wild type for allele B. But the overlapping peak of C and T is present. Allele “A” cannot be read due to dirty sequence at the end. |
|  | 143 | Pink left | 14/02/12: A8 | Wild type for allele A and B. Read is quite impressive and both alleles can be read. |
|  | 149 | Pink left | 14/02/12: B8 | Wild type for allele A and B. Read is quite impressive and both alleles can be read. |
|  | 150 | Pink left | 14/02/12: C8 | Wild type for allele A and B. Read is quite impressive and both alleles can be read. |
